# Visual search guidance uses coarser template information than target-match decisions

**DOI:** 10.3758/s13414-022-02478-3

**Published:** 2022-04-26

**Authors:** Xinger Yu, Simran K. Johal, Joy J. Geng

**Affiliations:** 1 Center for Mind and Brain, University of California, 267 Cousteau Pl, Davis, CA 95618 USA; 2 Department of Psychology, University of California, One Shields Ave, Davis, CA 95616 USA

**Keywords:** Visual search, Target template, Attentional guidance, Target-match decisions

## Abstract

When searching for an object, we use a target template in memory that contains task-relevant information to guide visual attention to potential targets and to determine the identity of attended objects. These processes in visual search have typically been assumed to rely on a common source of template information. However, our recent work (Yu et al., 2022) argued that attentional guidance and target-match decisions rely on different information during search, with guidance using a “fuzzier” version of the template compared with target decisions. However, that work was based on the special case of search for a target amongst linearly separable distractors (e.g., search for an orange target amongst yellower distractors). Real-world search targets, however, are infrequently linearly separable from distractors, and it remains unclear whether the differences between the precision of template information used for guidance compared with target decisions also applies under more typical conditions. In four experiments, we tested this question by varying distractor similarity during visual search and measuring the likelihood of attentional guidance to distractors and target misidentifications. We found that early attentional guidance is indeed less precise than that of subsequent match decisions under varying exposure durations and distractor set sizes. These results suggest that attentional guidance operates on a coarser code than decisions, perhaps because guidance is constrained by lower acuity in peripheral vision or the need to rapidly explore a wide region of space while decisions about selected objects are more precise to optimize decision accuracy.

**Supplementary Information:**

The online version contains supplementary material available at 10.3758/s13414-022-02478-3.

## Introduction

When looking for an object, we engage in a continuous look-and-identify cycle in which we use target information in memory (i.e., the target or attentional template) to guide eye-movements to probable targets and then make decisions about the match (Desimone & Duncan, 1995; Duncan & Humphreys, 1989; Wolfe, 2021). These processes are typically assumed to rely on the same information from a single target template. However, our recent work (Yu et al., 2022) found that the precision of attentional guidance and target-identity decisions differed when searching for a target amongst linearly separable distractors (e.g., an orange target amongst yellower distractors). Real-world search, however, rarely involves linearly separable distractors. Therefore, in the current studies, we test if attentional guidance uses a “fuzzier” version of the target template compared with target decisions during more typical visual search conditions.

Most models of visual search (Bundesen, 1990; Desimone & Duncan, 1995; Wolfe, 2021) include the concept of the attentional template (Duncan & Humphreys, 1989). It refers to an internal representation of target information held in working or long-term memory during visual search (Carlisle et al., 2011; Woodman et al., 2013). Activated shortly before the search task (Grubert & Eimer, 2018), the target template guides selective attention towards objects with template-matching attributes (Eimer, 2014) and is used to decide if the object is a target-match (Cunningham & Wolfe, 2014). Attentional guidance towards template-similar objects is presumed to occur because information in the target template is used to modulate sensory gain (Reynolds & Heeger, 2009). For example, when looking for a red colored object, it is assumed that the sensory gain of neurons that preferentially encode “red” anywhere in the visual field is enhanced (Andersen et al., 2008; Liu et al., 2007; Treue & Trujillo, 1999).

Once attention selects a candidate object, a decision must be made regarding the exact identity of the stimulus as a target-match or non-match (Castelhano et al., 2008; Rajsic & Woodman, 2020). This decision is a time-consuming portion of the look-identify cycle and must be accurate if visual search is to be ultimately successful. Therefore, more precise attentional templates are expected to improve visual search efficiency by enhancing attentional guidance to the correct targets and by accelerating target-match decisions (Hout & Goldinger, 2015; Malcolm & Henderson, 2009, 2010).

Although attentional guidance and match decisions are often hypothesized to rely on the same template information, Wolfe (2021) recently argued that the search template should be separated into two: a “guiding” template in working memory that is used to deploy attention to potential targets; and a “target” template in long-term memory that is used to determine if a candidate object is the target. For example, when looking for a blue coffee mug, your search will be guided to blue objects because color is a simple guiding feature, but once a blue object is selected, the more precise shape information in the target template will contribute to the identification of this blue object as the target or not. Consistent with the idea that guidance and identity decisions rely on different information from the target template, our recent study (Yu et al., 2022) provided evidence that when looking for an orange target that appears predictably amongst linearly separable (e.g., yellow) distractors, early attentional guidance is based on relational information (e.g., prioritizing the “reddest” object regardless of its exact hue) whereas subsequent match decisions are made against an “optimal” off-target feature (e.g., the slightly redder version of the orange target). Our findings suggest that attentional guidance operates on a coarser code to weight sensory information than target-match decisions, which uses more precise information to determine identity (Kerzel, 2019; Rajsic & Woodman, 2020).

**Fig. 1:**
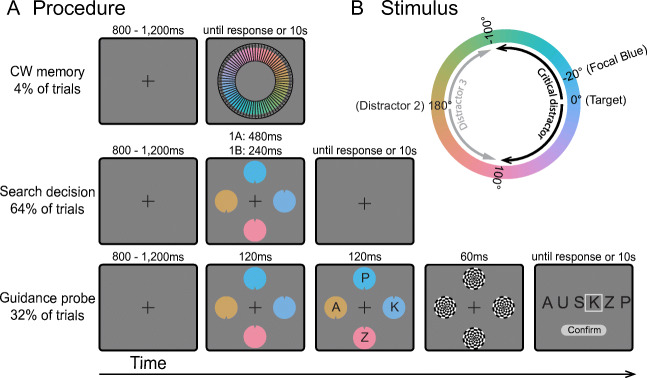
A) Example of *color wheel memory, search decision*, and *guidance probe* trials in Experiments 1A-B. *Color wheel memory* trials: Participants were instructed to type the number corresponding to the remembered target color. *Search decision* trials: Participants were instructed to locate the target color circle and report the position of the notch. Visual feedback was given immediately after the response. *Guidance probe* trials: Participants were instructed to report all letters on the response screen that they remembered seeing in the probe display. B) The color wheel used in both experiments. The illustrated target blue color (0°) was 20° rotated from the focal color within the blue color category. The black band indicates the range of colors used for critical distractors (-100° - 100°). The -20° critical distractor was the focal blue color. The second distractor was always 180° rotated from the target. The light gray band indicated the range of colors used for the last distractor (110° - 250°) (Color figure online)

The aim of the current experiments is to test if attentional guidance and target-match decisions for a target defined by a single feature (color) rely on different degrees of template precision during more common visual search conditions. The experiments involve randomly intermixing frequent *search decision* trials with infrequent *guidance probe* trials (Fig. 1A). On *search decision* trials, participants search for a predefined target-color circle and make a manual response to indicate the location of a notch inside the target. The outcome of search decision trials reflects the culmination of processes dedicated to the allocation of attention to potential targets and decisions regarding the identity of target. While reaction times would reflect all of the processes contributing to the final decision, the error rates are a direct measurement of the frequency with which a distractor is misidentified as the target in the *final binary decision*. On *guidance probe* trials, the search array is initially displayed just as on search decision trials, but then a letter rapidly appears inside each search stimulus (Gaspelin et al., 2015; Kim & Cave, 1995). Then, the entire array disappears, and participants are asked to recall as many letters as possible. The probability that the probe letter at a given location is reported indexes *initial attentional guidance* because participants will be more likely to report the letter at locations selected by spatial attention at the time the letter probes appear (Gaspelin & Luck, 2018). If guidance uses coarser template information than decisions, then participants will report letter probes on a wider range of distractors than those that are ultimately misidentified as targets. Alternatively, if a single fixed representation is used at the two stages, we expect the range of distractors that capture attentional guidance early on to be the same as the range of distractors that will be misidentified as the target.

## Experiments 1A-B

### Method

#### Participants

To determine the sample size for Experiment 1, we first conducted a pilot study with 32 participants (data were not included in Experiment 1) using similar methods and procedures in Experiment 1A. The smallest effect size of the two dependent measures of interest (in this case the probability of reporting the probe letter at critical distractor location, *d* = 0.393) was entered into G*power calculation (http://www.gpower.hhu.de/). The results estimated that *N* = 70 was necessary to detect significant effects (*p* = .05, two-tailed) with a power .9. Data were collected online using the Testable platform (https://www.testable.org/) until we obtained a sample of 70 participants after exclusion criteria were applied. 45 participants in Experiment 1A and 47 participants in Experiment 1B were excluded from the analysis because of poor performance in search decision trials (accuracy in decision trials with far critical distractors (±50° to ±100°) was below 80%) and insufficient guidance probe trials (the number of effective guidance trials was below 80%; see Statistical Analysis section for which guidance trials were excluded from the analysis). A large number of outliers was expected due to the fact that the experiment was conducted online through SONA and course credit was not tied to performance. 140 participants (Experiment 1A: *N* = 70, self-reported 12 males, self-reported 58 females, 2 left-handed, ages from 18 – 26 years; Experiment 1B: *N* = 70, self-reported 14 males, self-reported 56 females, 7 left-handed, ages from 18 – 43 years) from University of California, Davis participated online in Experiment 1 in partial fulfillment of a course requirement. A given participant completed only one experiment (Experiment 1A or 1B). Each participant provided written informed consent in accordance with the local ethics clearance as approved by the National Institutes of Health. Each participant’s color vision was assessed by self-report. All participants had normal or corrected-to-normal vision, and all had normal color vision.

#### Stimuli

All experiments were conducted online through Testable. All stimuli were created in Illustrator, saved as PNG files, and uploaded to Testable.org. All stimuli were presented against a gray background (color hue = ‘#808080’). The target and distractor colors were selected from a color wheel defined in LAB color space (a, b coordinates = 0, 0; luminance = 70; from Bae et al., 2015). The color wheel was an approximation to the cited color space as individual monitors were unable to calibrate in the online experiments. Two target colors (218°, 258°) were counterbalanced across participants. Each participant was assigned a single target color throughout the experiment. We used the colors that were ±20° rotated from the focal blue color (i.e., the best representative of the blue color category) as the target colors (Fig. 1B). Non-focal colors were used to assess if the expected memory bias for the target color towards the category center would also be present in visual search guidance and decisions (Bae et al., 2015; Nako et al., 2016). Two target colors were used to ensure that our results were not due to spurious effects associated with one color and yet minimize noise in perception due to uncontrolled color variation caused by participants’ environments (e.g., monitors, graphic cards, screen specifications, and lighting conditions). Because the target colors did not affect performance (Experiment 1A: *ps* > .13; Experiment 1B: *ps* > .06), the data were collapsed in all subsequent analyses to maximize statistical power. For descriptive simplicity, the target color will always be referred to as +20° rotations from the focal blue color. The experiments included three types of trials: 1) *color wheel memory* trials to measure the template content in long-term memory and independent of simultaneous distractor competition; 2) *search decision* trials to assess how target templates are used during the target decision making stage of visual search; 3) *guidance probe* trials to test how target templates are used during the initial guidance stage of visual search.

The color wheel in the *memory* trials (Fig. 1A) was divided into 72 bins (5° per bin) and each bin had a number attached. Participants reported the number of the color wedge that best matched the target color in memory. There was a total of six possible rotations of the color wheel. *Search decision* trials were composed of a target and 3 distractor circles (radius: 50 pixels), evenly arrayed around a virtual circle with a radius of 350 pixels (Fig. 1A). The first distractor (referred to as the “critical distractor”) was constructed in steps of 10° from the target color to ±100° rotations from the target color, resulting in a total of 20 distractor colors (Fig. 1B). Among the 20 colors, the -20° distractor was the focal blue color (Fig. 1B) and served to interrogate the response bias towards the category center. The second distractor color was always rotated 180° from the target color and was expected to interfere very little with target selection (Fig. 1B). The color of the last distractor changed on a trial-by-trial basis and was selected from the rest of the color wheel (110° - 250°) in steps of 10° in order to inject visual variability in the search display (Fig. 1B). To vary the absolute positions of objects, the search array was randomly rotated 40° clockwise or counterclockwise along an imaginary circle on every trial. Each search item had a small notch that appeared at the top or bottom. The notch on the target and the critical distractor appeared equally often on the top and bottom (50% each). The position of the notch on the target and the critical distractor was the same on 50% of trials. The notch positions of the two non-critical distractors varied randomly with the constraint that amongst the four objects, there were always two with notches at the top and two with notches at the bottom on every trial. On *guidance probe* trials (Fig. 1A), an uppercase letter in the English alphabet was presented in white Arial typeface at the center of each search item. The font size of letters (15pt) was set to be very small to encourage participants to move their eyes to identify the letter. The letters on a given trial were selected at random, without replacement, from a letter list composed of Q, W, E, U, P, A, J, L, Z, and M. A subsequent response screen displayed six letters in white, including the four presented in the previous probe displays and two fillers randomly chosen from the letter list.

#### Design

Participants completed 16 practice trials composed of all three types of trials. The main experiment was composed of 12 color wheel memory trials, 160 search decision trials and 80 guidance probe trials. Trials were presented in 80 mini-blocks, each containing 1-3 decision trials and 1 guidance trial. The color wheel memory trials were presented randomly, with the constraint that there could never be two consecutive memory trials.

#### Procedure

An example of the target color was presented prior to the beginning of the experiment. On *color wheel memory* trials (4% of trials), participants were required to type the number of the color wedge that best matched the target color in memory in a response box at the bottom of the screen. The color wheel remained on the screen until response. On *search decision* trials (64% of trials), the search array appeared on the screen for 480ms in Experiment 1A. Upon presentation of the display, participants searched for the predefined target-color circle and reported the notch position by pressing the keyboard button “O” for top and button “K” for bottom. Visual feedback was provided immediately following the response. On *guidance probe* trials (32% of trials), the search array was presented for 120ms, followed immediately by a letter superimposed on each search item for 120ms (the letter-probe array) (Gaspelin & Luck, 2018). Next, the search array was replaced with circular checkerboard masks (radius: 50 pixels) for 60ms, which served to minimize shifts of attention within iconic memory after the probe array disappeared (Loftus & Shimamura, 1985). Finally, the response display screen appeared until response. Participants used the mouse to choose all probe letters on the response screen that they remembered seeing on the probe display. Participants clicked on zero to four letters: a white box surrounded the letter when it was selected. They pressed “confirm” when selection was complete. Because guidance trials were fewer and randomly interleaved between decision trials, participants could not anticipate the letter probe task. Thus, the letters perceived should only be those on objects that early guidance had been able to select within the first 120ms search display based on the target template (i.e., color) in a “winner-take-all” competition. Furthermore, the short duration of the letter display was chosen to maximize the likelihood of permitting only one shift of attention on most trials (see Statistical Analysis section for evidence of this). If no response was recorded within 10s, all three types of trials automatically terminated. After response, a central fixation cross was presented for 800-1200ms before the next trial started. Participants were instructed to fixate on the center cross until task stimuli were presented.

Because target identification requires the accumulation of perceptual evidence, we presented the *search decision* trials for longer than the *guidance probe* arrays in Experiment 1A. However, this design allows for the possibility that observed differences between the two trial types are due to differences in display duration. To control for this possibility, Experiment 1B was identical to 1A except that the exposure duration of the search displays on *decision* trials was shortened to 240ms. If longer display durations are necessary for decision processes to be more precise than guidance, then there should be no differences between the precision of guidance and decisions in this experiment. However, if decisions are still more precise than guidance with shorter display durations, the results would indicate that the information underlying attentional guidance vs. target decisions is inherently different.

#### Statistical analysis

The color wheel in the *memory* task was composed of 72 color wedges sampling color hues in steps of 5°. Therefore, the relative click distance from the veridical target color, which reflects the degree of error in the reported color, was divided into 5° bins (Fig. 3A). Trials where the response errors were beyond ±60° were removed from data analyses, which accounted for 0.60% data in Experiment 1A and 0.48% data in Experiment 1B. The distribution of color wheel clicks was then fitted with a Gaussian function (Fig. 3A). This resulted in the estimation of parameter μ_mem_ (mean of the color wheel click frequency distribution), which corresponds to the central tendency in the color judgments, and the estimation of parameter σ_mem_ (standard deviation of the click frequency distribution), which corresponds to the precision of color judgments where smaller values indicate higher precision.

*Search decision* trials with an RT less than 250ms or greater than 2500ms were also excluded from the analyses, which resulted in 1.11% and 0.70% of the decision trials being dropped in Experiment 1A and 1B, respectively. When analyzing error rates on decision trials, we exclusively analyzed trials where the notch position of the critical distractor and that of the target were opposite, i.e., “notch-opposite” trials (see Supplemental Materials for full description of error rates). The notch positions of the two non-critical distractors on these trials were also opposite. Thus, if one of the two non-critical distractors was selected as the target, the error would have an equal probability of being coded as a "target correct" or a "critical distractor error"; however, such errors were rare (see Fig. S1 in Supplemental Materials). The majority of errors were due to selection of the critical distractor and therefore errors on these “notch-opposite” trials were used as an estimate of the probability that participants misidentified the critical distractor as the target. We calculated the frequency of misidentifications attributed to selection of each critical distractor for each participant as a metric of decision precision. For example, if the participant made 9 out of 10 errors on trials with the -10° critical distractor, we calculated the frequency of identification errors for the -10° distractor as .9. This calculation was made for every critical distractor separately. We then fitted the error frequencies with a Gaussian function. This resulted in the estimation of parameter μ_dec_ (mean of the decision error frequency distribution), which corresponds to the central tendency of match decisions, and the estimation of parameter σ_dec_ (standard deviation of the error frequency distribution), which corresponds to the precision of decision process.

**Fig. 2:**
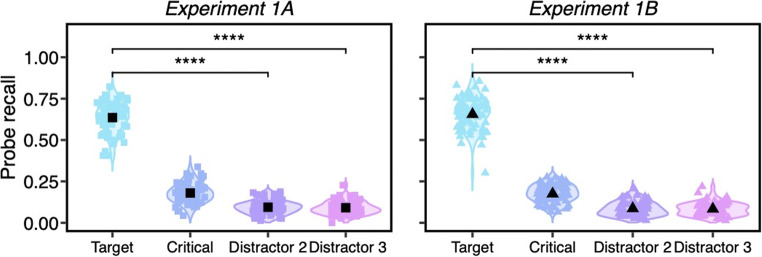
The percentages of probe letters reported at the target, critical distractor and non-critical distractor (Distractor 2 and Distractor 3) locations on *guidance probe* trials. Trials included in these analyses were ones in which only one letter was reported, and the letter was present in the probe array, which represented 88% or more of the guidance probe trials. The colored dots represent individual data points, and the black ones indicate the mean values. All error bars are the 95% confidence intervals (Color figure online)

*Guidance probe* trials were discarded if more than one probe letter was recalled. This resulted in a loss of 0.97% guidance trials in Experiment 1A and 1.51% guidance trials in Experiment 1B. These trials were excluded because we wished to only measure the first object that participants attended. Report of multiple probe letters precluded the ability to know which object was attended first. In addition, 9.79% trials in Experiment 1A and 9.71% trials in Experiment 1B were discarded when the reported letters were not present in the probe array. As shown in Fig. 2, probe letter recall was higher at the target location (*M*_*1A*_ = 63.57%, CI_1A_ = [61.42% 65.72%]; *M*_*1B*_ = 65.56%, CI_1B_ = [63.26% 67.87%]) than at the non-critical distractor locations (Distractor 2: *M*_*1A*_ = 9.39%, CI_1A_ = [8.43% 10.36%], *M*_*1B*_ = 8.61%, CI_1B_ = [7.58% 9.65%]; Distractor 3: *M*_*1A*_ = 9.07%, CI_1A_ = [8.11% 10.03%], *M*_*1B*_ = 8.34%, CI_1B_ = [7.41% 9.28%]), *ts* > 36.13, *ps* < .0001, *ds* > 4.31, BF_10_ > 1,000. This demonstrates that the probe task is a sensitive measure of attentional allocation to individual items. The percentage of trials in which only the probe letter at the critical distractor location was reported was used as the index of initial attentional guidance to the critical distractor. Consistent with search decision trials, we fitted a Gaussian function to the reported frequency of letters on critical distractors. This resulted in the estimation of parameter μ_gui_ (mean of the guidance probe recall frequency distribution), which corresponds to the central tendency of attentional guidance, and the estimation of parameter σ_gui_ (standard deviation of the recall frequency distribution), which corresponds to the precision of initial guidance.

**Fig. 3:**
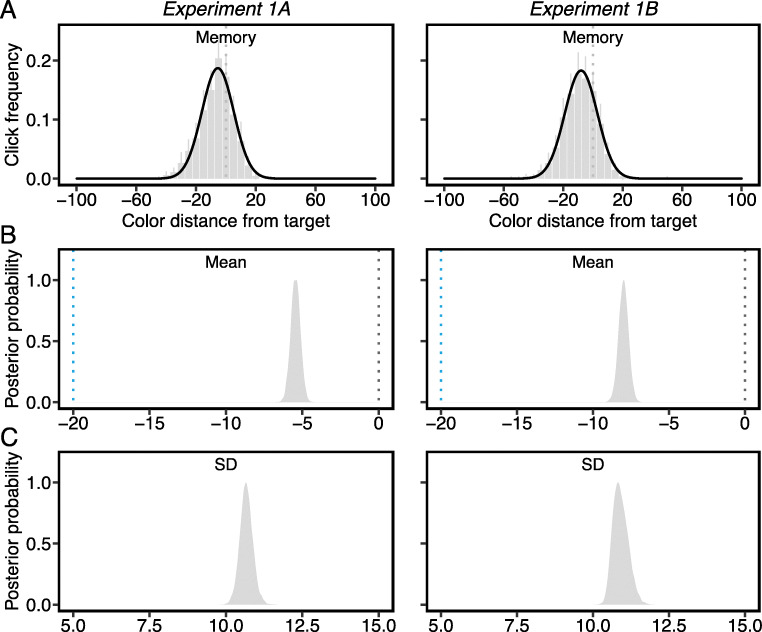
A) Group averages of click distance from the target color in the color wheel memory task. Raw data divided into 5° bins. Black solid lines are Gaussian distribution fits. All error bars are the 95% confidence intervals. B) Posterior distribution of μ_mem_ values from Gaussian fits. The gray dotted lines indicate the true target color (0°), and the blue lines indicate the focal blue color (-20°) at the category center. C) Posterior distribution of σ_mem_ values from Gaussian fits

All parameters from the Gaussian functions were estimated using a hierarchical Bayesian parameter (HBA) estimation method. To perform HBA, we used the R package, Bayesian Regression Models using ‘Stan’ (brms) (Bürkner, 2017, 2018) and the probabilistic programming language Stan (Carpenter et al., 2017). Normal and Gamma distributions were used to set the hyper priors of the normal mean (μ ~ Normal (0, 1)) and standard deviation (σ ~ Gamma (5, 1)). Given the small number of data points per participant (due to constraints in online experimentation), we only estimated the group parameter values to capture commonalities across individuals. Each chain was run with 5000 samples, with the first 2500 warm-up samples discarded as burn-in. A total of 4 chains were run, leading to 10,000 total posterior samples. Convergence was assessed by computing the Gelman-Rubin Ȓ statistic for each parameter. The range of R̂ values across all group parameter estimates was between 0.99-1.05, suggesting satisfactory convergence. Goodness of fit was visually inspected with the posterior predictive check method (Figs. 3 and 5). For each posterior distribution, we reported the mean posterior estimates and 95% credible intervals. Because all parameters were estimated with a hierarchical Bayesian approach, we conducted hypothesis testing directly on the posteriors rather than relying on frequentist statistics. For example, to assess whether μ_mem_ was significantly more negative than 0°, indicating a memory bias towards the color category center (i.e., the most typical color exemplar of the target category), we report the probability of posterior values being less than zero (Fig. 3B).

In addition to modeling the responses with the Gaussian function, we also directly compared the standard deviation of each participant’s recall (SD_gui_) and error (SD_dec_) responses using a paired sample *t* test. This analysis is complementary to the analysis of σ_gui_ and σ_dec_ from the Gaussian distribution. In contrast to σ_gui_ and σ_dec_, which are estimated from the pooled data across individuals, this analysis considers the within subject variance but is a less precise measurement of an individual’s attentional guidance and match decisions (due to the small number of data points per participant).

## Results

### Analysis of group frequency distributions with the Gaussian function using Bayesian statistics

#### Analysis of the contents of the target template in memory

The distributions of color wheel click on memory trials were estimated by fitting the Gaussian function (Fig. 3A). We found significantly negatively shifted μ_mem_ values (Fig. 3B) in both experiments (*M*_*1A*_ = -5.45°, CI_1A_ = [-6.05° -4.84°]; *M*_*1B*_ = -7.99°, CI_1B_ = [-8.63° -7.36°]), probability > .99, suggesting that colors exhibited memory biases towards the category center (focal blue color: -20°) (Bae et al., 2015; Hardman et al., 2017). Additionally, the estimated σ_mem_ (Fig. 3C) in both experiments were around 10° (*M*_*1A*_ = 10.67°, CI_1A_ = [10.25° 11.10°]; *M*_*1B*_ = 10.91°, CI_1B_ = [10.49° 11.44°]), indicating that the memory representation of the target was very precise. If this target template in memory was used to generate target-match decisions and/or guide attention (Yu et al., 2022), we expect the precision of those processes to match the precision of the target representation in memory.

#### Analysis of the precision of attentional guidance and match decisions

**Fig. 4:**
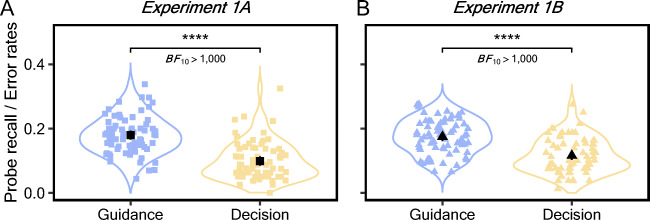
The percentages of letters reported on *critical distractors* on guidance probe trials and the error rates on search decision trials. The two measures provide information about the likelihood of attending to critical distractors and misidentifying them as the target. The colored dots represent individual data points, and the black ones indicate the mean values. All error bars are the 95% confidence intervals (Color figure online)

To test our main hypothesis that guidance would be less precise than decisions, we first directly compared percentages of letters reported on critical distractors on guidance probe trials and error rates on search decision trials using a paired *t* test (Fig. 4). The two measures provide information about whether critical distractors attracted initial attention and whether they were eventually misidentified as targets. The probe letter recall on critical distractors (*M*_*1A*_ = 17.97%, CI_1A_ = [16.56% 19.37%]; *M*_*1B*_ = 17.48%, CI_1B_ = [16.20% 18.75%]) was significantly higher than the decision error rate (*M*_*1A*_ = 9.90%, CI_1A_ = [8.48% 11.33%]; *M*_*1B*_ = 11.60%, CI_1B_ = [10.30% 12.89%]) (Experiment 1A: *t*(69) = 8.76, *p* < .0001, *d* = 1.04, BF_10_ > 1,000; Experiment 1B: *t*(69) = 7.45, *p* < .0001, *d* = 0.89, BF_10_ > 1,000). Moreover, comparisons between probe letter recall and error rates remained significant when each subtracted from chance levels of response (probe recall: 16.67%; error rates: 50%), Experiment 1A: *t*(69) = 44.70, *p* < .0001, *d* = 5.37, BF_10_ > 1,000, Experiment 1B: *t*(69) = 49.65, *p* < .0001, *d* = 5.93, BF_10_ > 1,000.

**Fig. 5:**
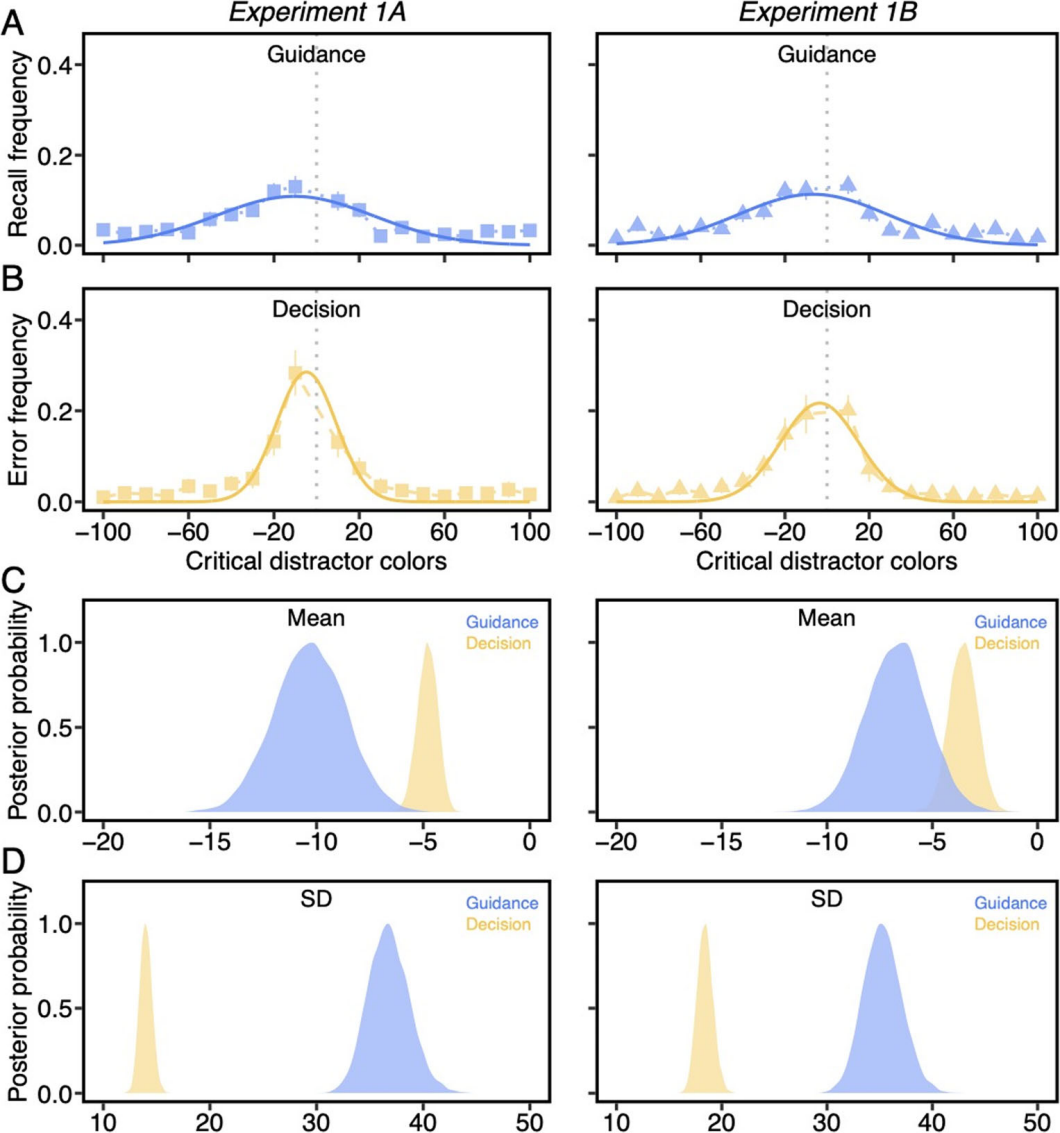
A) The frequency with which letters were recalled on each critical distractor. B) The frequency with which each critical distractor was misidentified as the target. Solid curved lines are Gaussian distribution fits. The gray dash line indicates the true target color. All error bars are the 95% confidence intervals. C) Posterior distribution of μ values from Gaussian fits. D) Posterior distribution of σ values from Gaussian fits (Color figure online)

Next, we examined the frequency of letter recall (Fig. 5A) and decision errors (Fig. 5B) as a function of *each* critical distractor condition and fitted the response frequencies with the Gaussian distribution. First, both μ_gui_ (*M*_*1A*_ = -10.32°, CI_1A_ = [-13.62° -7.08°]; *M*_*1B*_ = -6.70°, CI_1B_ = [-9.59° -3.83°]) and μ_dec_ (*M*_*1A*_ = -4.80°, CI_1A_ = [-5.76° -3.92°]; *M*_*1B*_ = -3.53°, CI_1B_ = [-4.82° -2.23°]) were significantly negatively shifted (Fig. 5C), probability > .99, suggesting that the two subprocesses of visual search were both biased towards the category center. The memory bias for the target color measured from the color wheel memory task was present in both guidance and decisions, consistent with the notion that the target template is encoded in memory and used as the source information for visual search guidance and decisions.

To assess whether a wider range of critical distractors attracted attention than those that were misidentified as the target, we compared the σ values estimated from the fitted Gaussian distributions, which serve as the statistical analogue for the magnitude of precision. As can be seen from the nonoverlapping posteriors (Fig. 5D), σ_gui_ (*M*_*1A*_ = 36.78°, CI_1A_ = [33.17° 40.80°]; *M*_*1B*_ = 35.28°, CI_1B_ = [31.83° 39.02°]) was significantly larger than σ_dec_ (*M*_*1A*_ = 13.99°, CI_1A_ = [12.91° 15.14°]; *M*_*1B*_ = 18.41°, CI_1B_ = [17.00° 19.96°]) in both experiments, probability > .99. The difference in σ values provide strong evidence in support of the hypothesis that the precision of attentional guidance is coarser than the precision of target-match decisions. Similarly, levels of imprecision in attentional guidance were replicated in a supplemental experiment that had a fewer number of guidance probe trials (see Experiment 1C in the Supplemental Material). This suggests that the cause of broader attentional guidance to target-similar stimuli is unlikely to be due to expectations regarding the likelihood of seeing a letter probe trial.

#### Comparisons of guidance and decisions against memory precision

Next, we compared the precision of the color wheel memory performance, which represents the upper limit in the precision of the template, against guidance and decisions. The precision of guidance (σ_gui_) was significantly poorer than memory (σ_mem_) in both experiments (Experiment 1A: *M*_*diff*_ = 26.11°; Experiment 1B: *M*_*diff*_ = 24.38°), probability > .99. This suggests that the cause of broader attentional guidance to target-similar stimuli is not due to imprecision in the underlying memory representation, but rather due to poor attentional selectivity (Kerzel, 2019). Although there was also a statistical difference between σ_dec_ and σ_mem_, probability > .99, the average difference was more modest, 3.33° in Experiment 1A and 7.50° in Experiment 1B. The larger difference in Experiment 1B was likely due to limitations in evidence accumulation following the shorter exposure duration of the search array. Together, the results indicate that the target decision process uses more precise template information compared to initial attentional guidance. Interestingly, both were less precise than the actual memory for the target itself. This suggests that the limiting factor in guidance and decision precision during visual search is related to temporal or visual pressures rather than the fidelity of the template memory itself.

## Analysis of individual standard deviations using frequentist statistics

The previous results demonstrated that response distributions for attentional guidance were broader than for attentional decisions. However, the σ_gui_ and σ_dec_ parameters from the Gaussian distribution were calculated from pooled data across individuals. Next, we directly compared the standard deviation of each participant’s recall (SD_gui_) and error (SD_dec_) responses using a paired sample *t* test. This analysis takes into account the within subject variance, but the estimates of an individual’s attentional guidance and match decisions are less precise (due to the small number of data points per participant), and therefore serves as a complement to the previous analysis.

Additional participants (four in Experiment 1A and one in Experiment 1B) were excluded from this analysis because they made less than one incorrect search decision trial. The paired sample *t* test was significant in both experiments, Experiment 1A: *t*(65) = 5.09, *p* < .0001, *d* = 0.63, BF_10_ > 1,000, Experiment 1B: *t*(68) = 6.04, *p* < .0001, *d* = 0.73, BF_10_ > 1,000. The results confirmed that the standard deviation on guidance trials (*M*_*1A*_ = 48.88°, CI_1A_ = [46.19° 51.56°]; *M*_*1B*_ = 45.68°, CI_1B_ = [43.45° 47.92°]) was larger than the standard deviation on decision trials (*M*_*1A*_ = 37.05°, CI_1A_ = [33.68° 40.42°]; *M*_*1B*_ = 35.62°, CI_1B_ = [32.68° 38.56°]).

**Fig. 6:**
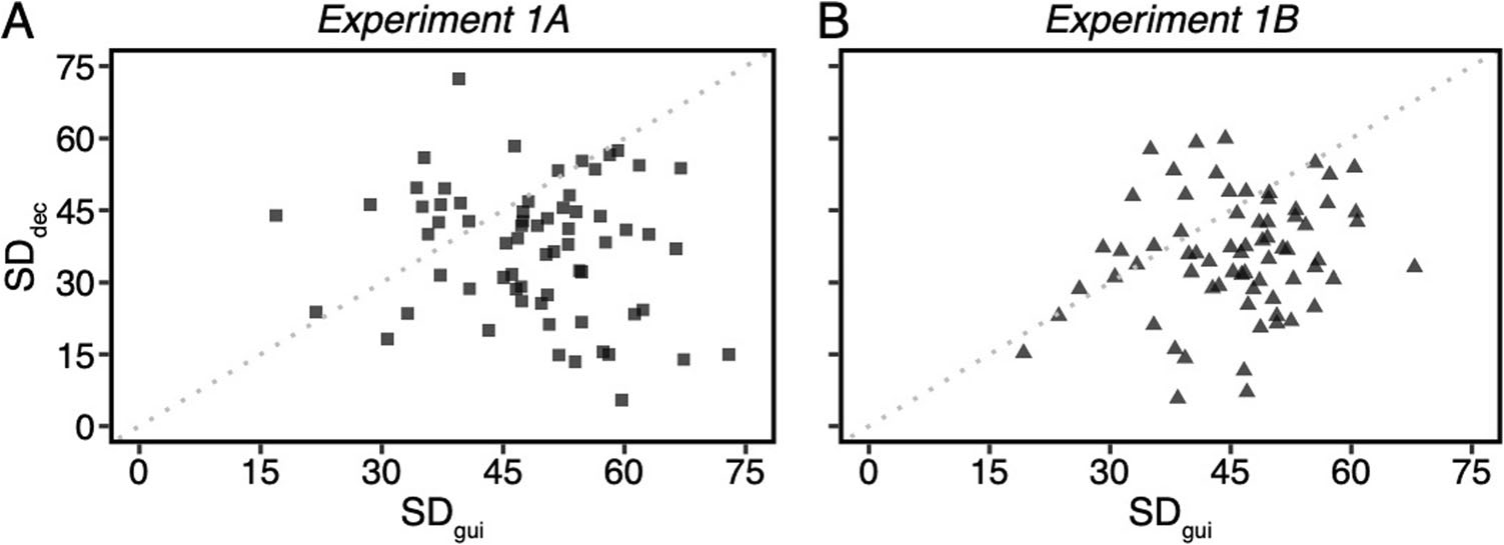
Scatterplot of each participant’s standard deviation of letter recall on guidance trials (SD_gui_, x axes) and error on search decision trials (SD_dec_, y axes). The black dots represent individual data points. The 76% data points in Experiment 1A and 77% data points in Experiment 1B are below the diagonal reference line (the gray dotted line), suggesting that most of individuals had larger SD_gui_ than SD_dec_ values

In order to visualize the relationship between the individual standard deviations on guidance trials versus decision trials, we plotted the two together. The scatterplot (Fig. 6) shows each participant’s standard deviation of letters recalled on guidance trials (SD_gui_, x axes) and errors on search decision trials (SD_dec_, y axes). Most of data points (76% in Experiment 1A and 77% in Experiment 1B) are below the diagonal reference line, indicating the majority of individuals had larger SD_gui_ than SD_dec_ values. This is consistent with the pooled Gaussian analyses above. These results provide strong convergent evidence in support of the hypothesis that the precision of attentional guidance is coarser than the precision of target-match decisions.

## Experiments 2A-B

The purpose of Experiment 2 was to provide a conceptual replication of Experiment 1 and test if imprecisions in attentional guidance will still occur on visual search trials with small set sizes. In this experiment, we modified the visual search paradigm to include a target and only one distractor. We predicted the precision of attentional guidance would increase but still be worse than the precision of target-match decisions.

### Participants

195 new participants from University of California, Davis participated online in Experiment 2A and 2B in partial fulfillment of a course requirement. 55 subjects were excluded by the same criteria in Experiment 1, which led to a total of 140 undergraduates (self-reported 22 males, self-reported 118 females, 11 left-handed, ages from 18 – 39 years). A given participant completed only one experiment (Experiment 2A or 2B). Each participant provided written informed consent in accordance with the local ethics clearance as approved by the National Institutes of Health. Each participant’s color vision was assessed by self-report. All participants had normal or corrected-to-normal vision, and all had normal color vision.

### Stimuli, design, Procedure & Statistical Analysis

**Fig. 7:**
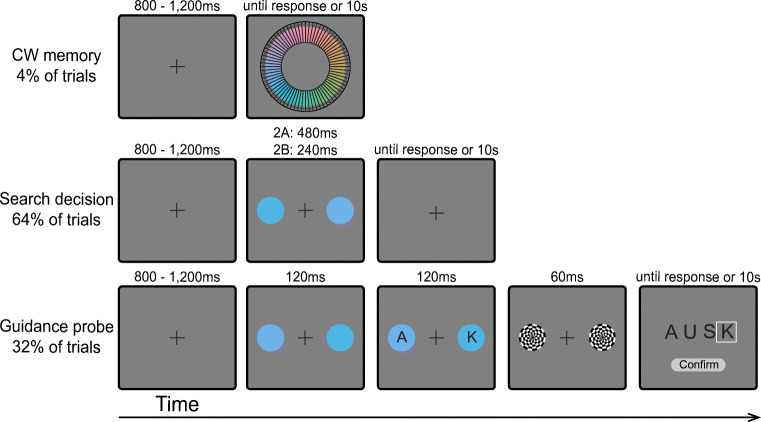
Example of *color wheel memory*, *search decision*, and *guidance probe* trials in Experiments 2A-B (Color figure online)

All aspects of Experiment 2 were identical to Experiment 1, with the following exceptions. *Search decision* trials (Fig. 7) consisted of two bilaterally presented target and critical distractor circles (distance between the center points: 350 pixels). Participants were instructed to indicate whether the target color appeared at the left side by pressing button “K” or at the right side by pressing button “L”. The stimuli appeared on the screen for 480ms in Experiment 2A and 240ms in Experiment 2B. *Guidance probe* trials (Fig. 7) started like search decision trials, but the search array appeared for only 120ms. Then, probe letters were superimposed on the search items for 120ms, after which all items disappeared. The letter list was the same as the list used in Experiment 1, except that the letter “L” was replaced with the letter “B” because “L” was now used for search trial responses. The subsequent response screen displayed four letters, including the two in probe displays and two fillers randomly chosen from the letter list. The two target colors were counterbalanced across participants and because there were no spurious differences (*ps* > .18), the data were collapsed in all subsequent analyses. Overall, 3.9% of trials in Experiment 2A and 3.9% of trials in Experiment 2B were removed from data analysis by the same criteria in Experiment 1.

## Results

### Analysis of group frequency distributions with the Gaussian function using Bayesian statistics

#### Analysis of the contents of the target template in memory

**Fig. 8:**
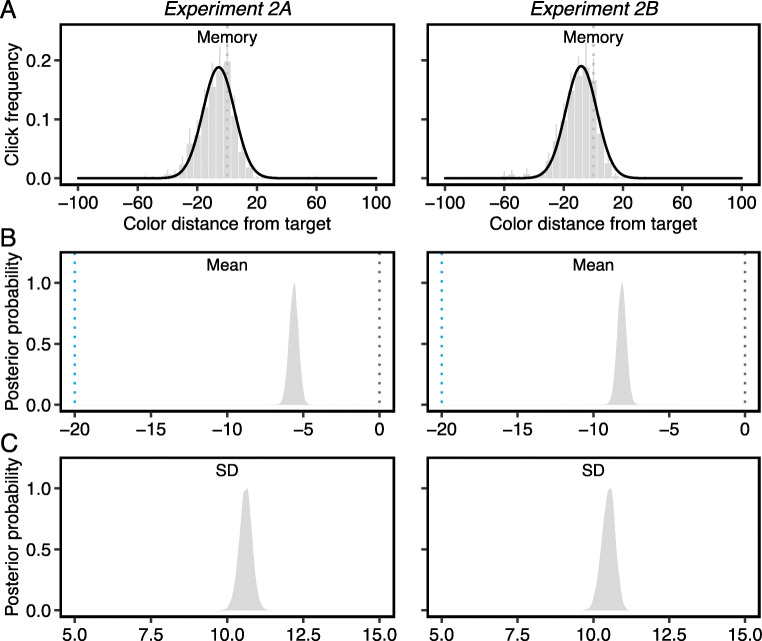
A) Group averages of click distance from the target color in the color wheel memory task. Raw data divided into 5° bins. Black solid lines are Gaussian distribution fits. All error bars are the 95% confidence intervals. B) Posterior distribution of μ_mem_ values from Gaussian fits. The gray dotted lines indicate the true target color (0°), and the blue lines indicate the focal blue color (-20°) at the category center. C) Posterior distribution of σ_mem_ values from Gaussian fits

The distributions of relative click distance on color wheel memory trials were fitted with the Gaussian function (Fig. 8A). The μ_mem_ values (*M*_*2A*_ = -5.63°, CI_2A_ = [-6.21° -5.05°]; *M*_*2B*_ = -8.13°, CI_2B_ = [-8.71° -7.55°]) were significantly negatively shifted (Fig. 8B), probability > .99. This result replicated those from Experiment 1, suggesting that the color memory is pulled towards the nearest category center. In addition, we found no difference in memory precision (*M*_*2A*_ = 10.60°, CI_2A_ = [10.18° 11.01°]; *M*_*2B*_ = 10.49°, CI_2B_ = [10.05° 10.89°]) between Experiment 1 and 2 (Fig. 8C), probability_1A>2A_ = .58, probability_1B>2B_ = .91, indicating that the stable search target is stored in long-term memory with high precision (Woodman et al., 2013) irrespective of whether it is used for four-item or two-item search.

#### Analysis of the precision of attentional guidance and match decisions

**Fig. 9:**
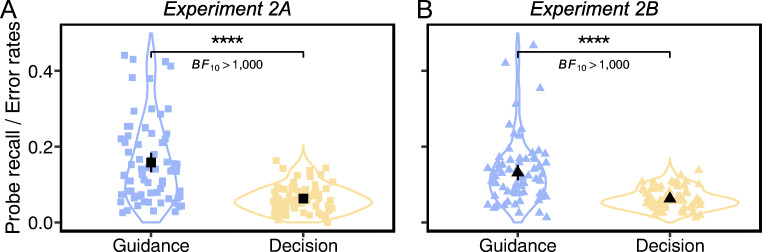
The percentages of letters reported on critical distractors on guidance probe trials and the error rates of selecting critical distractors as the target on search decision trials. The colored dots represent individual data points, and the black ones indicate the mean values. All error bars are the 95% confidence intervals (Color figure online)

A paired *t* test (Fig. 9) confirmed a significantly higher percentage of letters reported on critical distractors (*M*_*2A*_ = 15.82%, CI_2A_ = [13.25% 18.38%]; *M*_*2B*_ = 13.17%, CI_2B_ = [11.16% 15.18%]) than the error rate of selecting critical distractors as the target (*M*_*2A*_ = 6.32%, CI_2A_ = [5.46% 7.17%]; *M*_*2B*_ = 6.24%, CI_2B_ = [5.59% 6.89%]) (Experiment 2A, *t*(69) = 7.56, *p* < .0001, *d* = 0.90, BF_10_ > 1,000; Experiment 2B, *t*(69) = 7.22, *p* < .0001, *d* = 0.86, BF_10_ > 1,000). Comparisons remained significant when probe letter recall and error rates were subtracted from chance levels of response (probe recall: 25%; error rates: 50%), Experiment 2A: *t*(69) = 34.07, *p* < .0001, *d* = 4.07, BF_10_ > 1,000; Experiment 2B: *t*(69) = 41.99, *p* < .0001, *d* = 5.02, BF_10_ > 1,000. In replication of Experiment 1, participants were more likely to direct their attention to critical distractors, but easily reject them as nontargets.

**Fig. 10:**
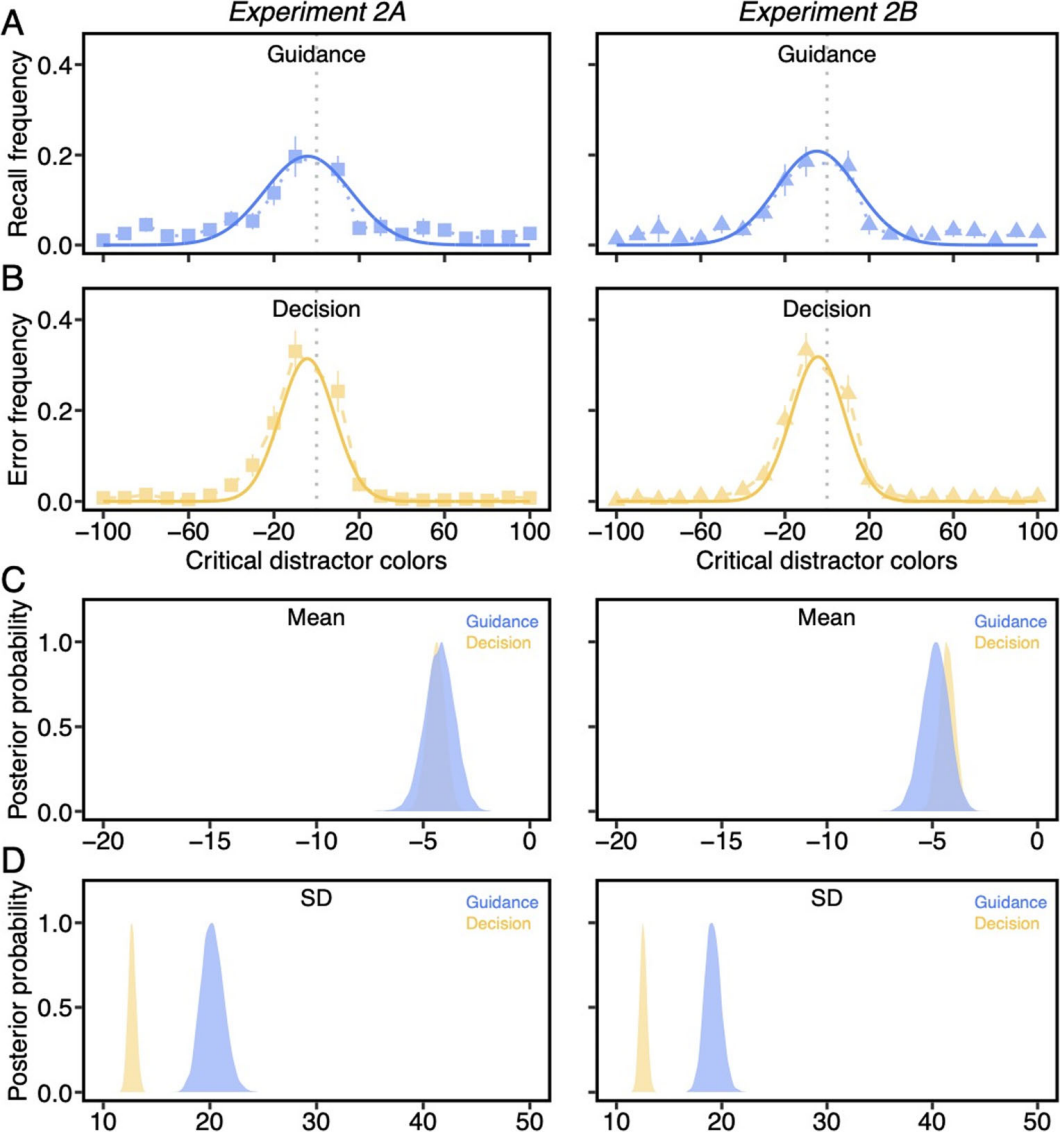
A) The frequency with which letters were recalled on each critical distractor. B) The frequency with which each critical distractor was misidentified as the target. Solid curved lines are Gaussian distribution fits. The gray dash line indicates the true target color. All error bars are the 95% confidence intervals. C) Posterior distribution of μ values from Gaussian fits. D) Posterior distribution of σ values from Gaussian fits (Color figure online)

We next computed the frequency of letter recall (Fig. 10A) and decision errors (Fig. 10B) as a function of *each* critical distractor condition and fitted the Gaussian function to frequency distributions in the same way as Experiment 1. First, both μ_gui_ (*M*_*2A*_ = -4.23°, CI_2A_ = [-5.62° -2.91°]; *M*_*2B*_ = -4.86°, CI_2B_ = [-6.15° -3.63°]) and μ_dec_ (*M*_*2A*_ = -4.39°, CI_2A_ = [-5.21° -3.61°]; *M*_*2B*_ = -4.31°, CI_2B_ = [-5.07° -3.57°]) were significantly negatively shifted (Fig. 10C), probability > .99, demonstrating a bias in guidance and decisions towards the category center. The shift in memory towards the category center was recapitulated in guidance and decisions, supporting the notion that a single memory template underlies both processes.

The comparisons of σ values, which were used to index the precision of guidance and decisions, showed that σ_gui_ (*M*_*2A*_ = 20.23°, CI_2A_ = [18.27° 22.41°]; *M*_*2B*_ = 19.14°, CI_2B_ = [17.67° 20.74°]) was significantly larger than σ_dec_ (*M*_*2A*_ = 12.70°, CI_2A_ = [12.01° 13.44°]; *M*_*2B*_ = 12.54°, CI_2B_ = [11.88° 13.21°]) in both experiments (Fig. 10D), probability > .99. This pattern converges with Experiment 1, suggesting that attentional guidance is a less precise process during visual search than match decisions. Furthermore, the σ_gui_ values (Experiment 1A - 2A: *M*_*diff*_ = 16.54°; Experiment 1B - 2B: *M*_*diff*_ = 16.14°) were much smaller, probability > .99, compared to Experiment 1, showing that the precision of attentional guidance improved substantially with smaller set sizes. In contrast, the set size effect on σ_dec_ (Experiment 1A - 2A: *M*_*diff*_ = 1.29°; Experiment 1B - 2B: *M*_*diff*_ = 5.87°) was significant but relatively weak, probability_1A>2A_ = .97, probability_1B>2B_ > .99.

*Comparisons of guidance and decisions against memory precision.* The σ_gui_ values were significantly greater than the σ_mem_ values (Experiment 2A: *M*_*diff*_ = 9.63°; Experiment 2B: *M*_*diff*_ = 8.65°), probability > .99, again suggesting that imprecise attentional guidance is not because of poor memory representations. In contrast, the average difference between σ_dec_ and σ_mem_ was only 2.10° in Experiment 2A and 2.04° in Experiment 2B, but statistically significant, probability > .99, highlighting the fact that the precision of decision process was closer to the precision of the target color held in long-term memory.

## Analysis of individual standard deviations using frequentist statistics

**Fig. 11:**
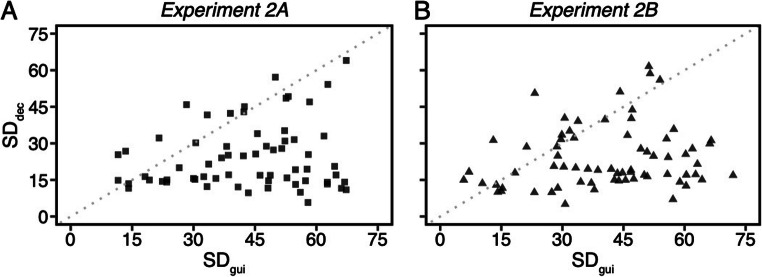
Scatterplot of each participant’s standard deviation of letter recall on guidance trials (SD_gui_, x axes) and error on search decision trials (SD_dec_, y axes). The black dots represent individual data points. The 85% data points in Experiment 2A and 78% data points in Experiment 2B are below the diagonal reference line (the gray dotted line), suggesting that most of individuals had larger SD_gui_ than SD_dec_ values

Additional participants (five in Experiment 2A and one in Experiment 2B) were excluded from this analysis because they made less than one incorrect search decision trial. The scatterplot (Fig. 11) shows each participant’s standard deviation of letters recalled on guidance trials (SD_gui_, x axes) and errors on search decision trials (SD_dec_, y axes). A paired sample *t* test confirmed that SD_gui_ (*M*_*2A*_ = 43.23°, CI_2A_ = [39.3° 47.16°]; *M*_*2B*_ = 40.67°, CI_2B_ = [36.74° 44.59°]) was significantly larger than SD_dec_ (*M*_*2A*_ = 24.38°, CI_2A_ = [21.08° 27.69°]; *M*_*2B*_ = 23.95°, CI_2B_ = [20.90° 27.00°]) in both experiments, Experiment 2A: *t*(64) = 7.99, *p* < .0001, *d* = 0.99, BF_10_ > 1,000, Experiment 2B: *t*(68) = 7.32, *p* < .0001, *d* = 0.88, BF_10_ > 1,000. This result is consistent with the σ values from the Gaussian distribution, suggesting that attentional guidance is a less precise process than match decisions.

## Discussion

The purpose of the current experiments is to test if attentional guidance is coarser than target-match decisions during visual search. To this end, we measured the likelihood of attentional guidance and misidentification decisions across a range of distractors from 10° up to 100° of separation from the target in color space. All trials began with identical displays for 120ms but then on a subset of trials (32% in Experiments 1 and 2, 16% in Supplemental Experiment 1C) the search display changed into a letter probe task followed by a mask. On these trials, participants reported any letters that they had seen. Despite this instruction, they reported only one letter on more than 98% of trials, suggesting that the timing of the experiment precluded the ability to shift attention to a second object after selecting the first object. The target letter was reported on about 65% of the time but that the critical distractor was also reported frequently on about 18% of trials in Experiment 1. This suggests that attentional guidance selected objects based on the target color within the first 120ms when participants still expected to find and identify the search target. Most importantly, the width of the guidance distribution was greater than that of identification errors. The results showed that a broader range of distractors capture initial attention than those that were ultimately misidentified as targets. Our findings provide evidence that template information operates at different scales of precision to guide attention and make identity decisions (Wolfe, 2021).

The target template has long been hypothesized to allocate attention to candidate objects by converting display-wide enhancement of template-matching features into spatially specific enhancement (Berggren et al., 2017; Eimer, 2014). However, recent research has found that target-similar cues that do not completely match the template contents also strongly capture attention (Kerzel, 2019), suggesting that attentional selection is imprecise compared to the memory template. In the current study, we found convergent evidence that the “tuning” of guidance was 8 ~ 26° broader than the target template in memory. In contrast, decisions about the identity of the target after a candidate object was selected were only 2° ~ 3° less precise than the template. The exception was in Experiment 1B when the search display was short and there were four items, suggesting that the precision of decision processes depends on sufficient time to accumulate perceptual evidence (Yu et al., 2022).

Together, these experiments suggest that attentional guidance and target-match decisions differ in precision during visual search. What could cause this difference between guidance and decisions? The low precision of attentional guidance is perhaps due to the need to rapidly prioritize attention to stimuli in peripheral vision where color and spatial acuity are poor (Hulleman, 2009; Rosenholtz, 2017). This would explain why the precision of the initial guidance improved substantially when the set size was smaller and there was less visual crowding (Experiment 2). The lower precision could also be due, in part, to internal noise in the target representation within the visual system or to a lower criterion to shift attention to an object given the low costs of selecting and rejecting a non-target item. In contrast, when making identity decisions, there is greater pressure for accuracy given the “high stakes” nature of identification, and more detailed information is available because the attended stimulus is in foveal vision (Castelhano et al., 2008; Rajsic & Woodman, 2020).

So far, our assumption has been that attentional guidance and decisions rely on the same source target representation but are constrained by differences in visual acuity (i.e., in peripheral vs. central vision) and perhaps response criterion. However, this is not incompatible with the possibility that there are two qualitatively different types of template information used at different stages of processing when the target object has multidimensional features (e.g., a blue, mug). For example, Wolfe and colleagues have argued that when looking for a large number of potential targets, search is guided by a “guiding template” in working memory that contains simple guiding features like color and orientation, and target identification, or object recognition, is determined by a precise “target template” in long-term memory (Cunningham & Wolfe, 2014; Wolfe, 2021). Depending on the processing stage at hand, the optimal feature to use might be different (e.g., use of “blue” for the guiding template and a specific shape for the “mug” decision). This is potentially because attentional guidance operates optimally based on basic features like color (Vickery et al., 2005; Wolfe et al., 2011), but these simple visual features for guidance towards an object may not be adequate to identify whether the attended object is the actual target. Because we only used a single color as the target throughout the experiment, our data cannot differentiate between all the ways in which information used for guidance might differ from decisions. Future work with multidimensional stimuli is necessary to flesh out how working and long-term memory representations of the target differ and are used to guide attention and make target decisions.

In conclusion, we used an attention-probe paradigm to compare the precision of attentional guidance and the precision of target-match decisions during visual search. Under different exposure durations and distractor set sizes, we consistently observed that guidance was coarser than match decisions. Our results offer a novel view of the search template that considers the unique demands of attentional guidance vs. decisions during visual search.

## Supplementary Information

ESM 1 (DOCX 413 KB)
