# Supplementary material for: Visual search guidance uses coarser template information than target-match decisions
Source: Atten Percept Psychophys. 2022 Apr 26;84(5):1432–45. doi: 10.3758/s13414-022-02478-3 (PMC9232460; doi:10.3758/s13414-022-02478-3)
Supplement: Supplementary file 1 — (DOCX 413 KB) [file 13414_2022_2478_MOESM1_ESM.docx]

**Supplemental material**

**Fig. S1** Error rates on *search decision* trials where the notch position of the critical distractor and that of the target were opposite or the same in Experiments 1A–B. Because the target and critical distractor had the same response on the “same” trials, the error rates reflect the likelihood of misidentifying one of the two noncritical distractors as the target. The error rates on these trials were low (*M*_1A_ = 3.79%, CI_1A_ [2.99%, 4.58%]; *M*_1B_ = 4.75%, CI_1B_ [3.82%, 5.68%]), suggesting that participants had a very low probability of selecting the noncritical distractors as the target. This supports the notion that error on “opposite” trials (on which the two noncritical distractors also had opposite notch positions) reflect the rate of erroneously selecting the critical distractor as the target

**Experiment 1C**

Our goal in Experiment 1C was to investigate whether imprecisions in attentional guidance were caused by expectations related to which trial type they might see, which should affect task switch costs. To that end, we increased the number of search decision trials and decreased the number of guidance probe trials. If coarser attentional guidance arises because of unexpected task switch costs (i.e., changing to the letter report task), then the precision of attentional guidance would significantly decline (or improve) when switches cannot be anticipated as well. On the other hand, if attentional guidance is inherently a less precise process, the precision of attentional guidance would remain the same, and be worse than the precision of target-match decisions.

**Method**

***Participants***

One hundred and five new participants from University of California, Davis, participated online in Experiment 1C in partial fulfillment of a course requirement. Thirty-five subjects were excluded by the same criteria in Experiment 1C, which led to a total of 70 undergraduates (self-reported 15 males, self-reported 53 females, self-reported two nonbinaries, seven left-handed, ages 18–29 years). Each participant provided written informed consent in accordance with the local ethics clearance as approved by the National Institutes of Health. Each participant’s color vision was assessed by self-report. All participants had normal or corrected-to-normal vision, and all had normal color vision.

***Stimuli, design, procedure, and statistical analysis***

All aspects of Experiment 1C were identical to those of Experiment 1A with the following changes. The main experiment was composed of 12 color wheel memory trials, 200 search decision trials and 40 guidance probe trials. Trials were presented in 40 mini-blocks, each containing one to nine decision trials and one guidance trial. Overall, 2.7% of trials in Experiment 1C were removed from data analysis by the same criteria in Experiment 1A.

**Results**

***Analysis of the contents of the target template in memory***

The distributions of relative click distance on color wheel memory trials were fitted with the Gaussian function (Fig. S2). The µ_mem_ values (*M_1C_* = −6.59°, CI_1C_ [−7.10°, −6.08°]) were significantly negatively shifted (Fig. S2), probability > .99. This result replicated those from Experiment 1A, suggesting that the color memory is pulled towards the nearest category center. In addition, the estimated σ_mem_ (Fig. S2) were around 10° (*M*_1C_ = 9.71°, CI_1C_ [9.27°, 10.18°]), again indicating that the memory representation of the target was very precise.

**Fig. S2 a** Group averages of click distance from the target color in the color wheel memory task. Raw data divided into 5° bins. Black solid lines are Gaussian distribution fits. All error bars are the 95% confidence intervals. **b** Posterior distribution of µ_mem_ values from Gaussian fits. The gray dotted lines indicate the true target color (0°), and the blue lines indicate the focal blue color (−20°) at the category center. **c** Posterior distribution of σ_mem_ values from Gaussian fits

***Analysis of the precision of attentional guidance and match decisions***

A paired *t* test (Fig. S3) confirmed a significantly higher percentage of letters reported on critical distractors (*M*_1C_ = 14.41%, CI_1C_ [12.85%, 15.96%]) than the error rate of selecting critical distractors as the target (*M*_1C_ = 10.18%, CI_1C_ [8.54%, 11.81%]), *t*(69) = 4.42, *p* = .0004, *d* = 0.53, BF_10_ = 534.18. Comparisons remained significant when probe letter recall and error rates were normalized against response chance level (probe recall: 16.67%; error rates: 50%), *t*(69) = 39.24, *p* < .0001, *d* = 4.69, BF_10_ > 1,000. In replication of Experiment 1A, participants were more likely to direct their attention to critical distractors, but easily reject them as nontargets.


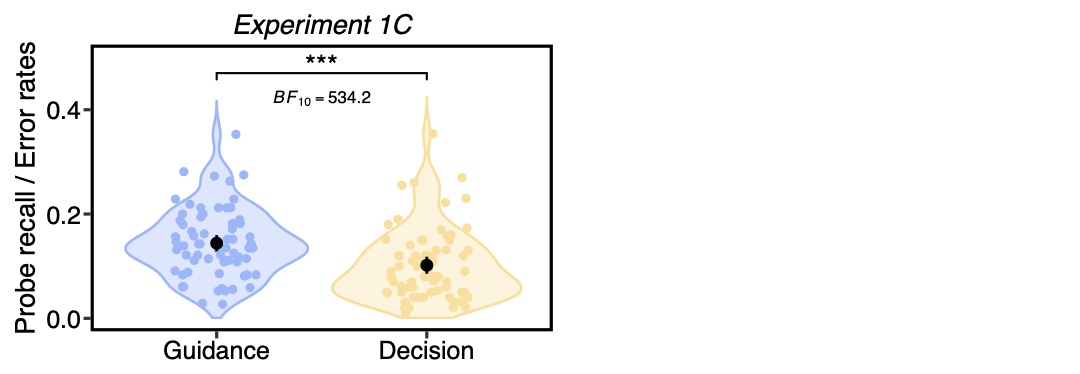


**Fig. S3** The percentages of letters reported on critical distractors on guidance probe trials and the error rates of selecting critical distractors as the target on search decision trials. The colored dots represent individual data points, and the black ones indicate the mean values. All error bars are the 95% confidence intervals

We next computed the frequency of letter recall and decision errors (Fig. S4) as a function of *each* critical distractor condition and fitted the Gaussian function to frequency distributions in the same way as Experiment 1A. First, both µ_gui_ (*M*_1C_ = −2.46°, CI_1C_ [−5.67°, 0.80°]) and µ_dec_ (*M*_1C_ = −2.61°, CI_1C_ [−3.46°, −1.82°]) were (marginally) significantly negatively shifted (Fig. S4), probability_gui_ = .93, probability_dec_ > .99, demonstrating a bias in guidance and decisions towards the category center. The shift in memory towards the category center was recapitulated in guidance and decisions, supporting the notion that a single memory template underlies both processes.


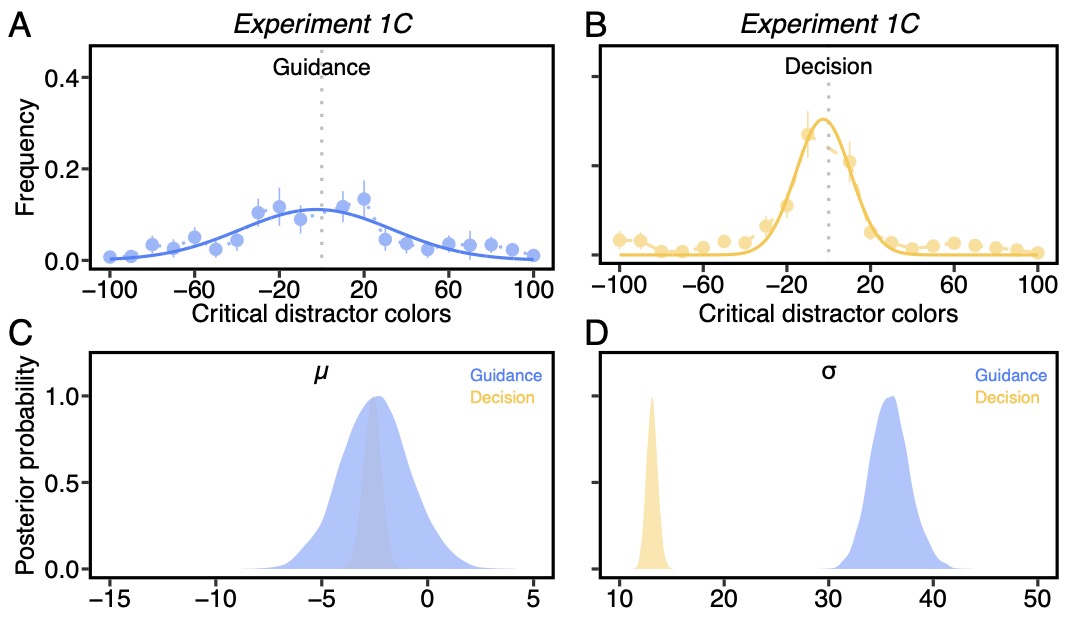


**Fig. S4 a** The frequency with which letters were recalled on each critical distractor. **b** The frequency with which each critical distractor was misidentified as the target. Solid curved lines are Gaussian distribution fits. The gray dash line indicates the true target color. All error bars are the 95% confidence intervals. **c** Posterior distribution of µ values from Gaussian fits. **d** Posterior distribution of σ values from Gaussian fits

The comparisons of σ values, which were used to index the precision of guidance and decisions, showed that σ_gui_ (*M*_1C_ = 35.88°, CI_1C_ [32.44°, 39.72°]) was significantly larger than σ_dec_ (*M*_1C_ = 13.11°, CI_1C_ [12.10°, 14.16°]), probability > .99. This pattern converges with Experiment 1A, suggesting that attentional guidance is a less precise process during visual search than match decisions. Furthermore, the σ_gui_ values were not significantly different (Experiment 1A–1C: *M*_diff_ = 0.92°), probability = .63, compared with Experiment 1A. This suggests that the cause of broader attentional guidance to target-similar stimuli is unlikely to be due to expectations regarding the likelihood of seeing a letter probe trial.

***Comparisons of guidance and decisions against memory precision***

The σ_gui_ values were significantly greater than the σ_mem_ values (*M*_diff_ = 26.17°), probability > .99, again suggesting that imprecise attentional guidance is not because of poor memory representations. In contrast, the average difference between σ_dec_ and σ_mem_ was only 3.40°, but statistically significant, probability > .99, highlighting the fact that the precision of decision process was closer to the precision of the target color held in long-term memory.
